# Supplementary material for: Biomarkers of collagen synthesis predict progression in the PROFILE idiopathic pulmonary fibrosis cohort
Source: Respir Res. 2019 Jul 12;20:148. doi: 10.1186/s12931-019-1118-7 (PMC6624898; doi:10.1186/s12931-019-1118-7)
Supplement: Supplementary file 1 — Table S1. Target and assay performance characteristics for the ELISA assays specific to each neoepitope. Table S2. Spearman correlations for baseline serum concentrations of synthesis neoepitopes in control and IPF subjects. Table S3. Unadjusted and adjusted (for baseline FVC and DLco) univariate analysis of overall survival in relation to 3-month change in neoepitope concentrations. Table S4. Spearman correlations for 3-month change in serum concentrations of synthesis and degradation neoepitopes in IPF subjects. Figure S1. Baseline comparison of collagen degradation neoepitope (BGM, C1M, C3M, C6M and CRPM) concentrations in healthy controls (n = 50) and participants with idiopathic pulmonary fibrosis (n = 145). Plots represent mean and 95% CI (error bars) adjusted for gender. Figure S2. Comparison of neoepitope concentrations in healthy controls (green) and participants with stable (blue) and progressive (red) idiopathic pulmonary fibrosis at baseline and subsequently at 1-, 3- and 6-months. Figure S3. Comparison of synthesis neoepitope concentrations adjusted for baseline FVC and DLco %predicted in healthy controls (green) and participants with stable (blue) and progressive (red) idiopathic pulmonary fibrosis at baseline and subsequently at 1-, 3- and 6-months. Figure S4. Comparison of degradation neoepitope concentrations adjusted for baseline FVC and DLco %predicted in healthy controls (green) and participants with stable (blue) and progressive (red) idiopathic pulmonary fibrosis at baseline and subsequently at 1-, 3- and 6-months. Figure S5. Comparison of neoepitope concentrations adjusted for baseline FVC and DLco %predicted in healthy controls (green) and participants with stable (blue) and progressive (red) idiopathic pulmonary fibrosis at baseline and subsequently at 1-, 3- and 6-months. (DOCX 1380 kb) [file 12931_2019_1118_MOESM1_ESM.docx]

**Biomarkers of collagen synthesis biomarkers predict progression in the PROFILE idiopathic pulmonary fibrosis cohort.**

Louise A Organ PhD^1^, Anne-Marie Duggan MSc^2^, Eunice Oballa MSc^2^, Sarah Taggart MSci^2^, Arthur R Kang’ombe^2^, Rebecca Braybrooke RGN^1^, Philip L Molyneaux MD PhD ^3,4^,Bernard North PhD^2^, Yakshitha Karkera^2^, Diana J Leeming PhD^5^, Morten A Karsdal PhD^5^, Carmel B Nanthakumar PhD^2^, William A Fahy MD^2^, Richard P Marshall MD PhD^2,^ R Gisli Jenkins MD PhD*^1^, Toby M Maher MD PhD*^3,4^

**Additional file 1**

| Assay name | Target | Antibody type | Detection range, LLOQ-ULOQ (ng/mL) | Intra-assay variation (%) | Inter-assay variation (%) | Assay principle reference |
| --- | --- | --- | --- | --- | --- | --- |
| C1M | MMP-2/9/13 degraded type I collagen | Monoclonal | 10-200 | 2.7-8.2 | 5.5-18.0 | (12) |
| C3M | MMP-9 degraded type III collagen | Monoclonal | 4-88 | 6.6-15.1 | 2.0-4.1 | (13) |
| C6M | MMP-2/9 degraded type vI collagen | Monoclonal | 6-267 | 2.0-8.0 | 4.0-18.0 | (32) |
| CRPM | MMP-1/9 degraded C-reactive protein | Monoclonal | 2.0-72.0 | 2.2-6.0 | 4.1-21.1 | (14) |
| BGM | MMP-9 degraded biglycan | Monoclonal | 4.1-190.0 | 2.0-6.0 | 5.0-20.0 | (33) |
| P1NP | N-terminal propeptide of type I collagen (formation marker) | Monoclonal | 14.0-516.0 | 1.0-8.0 | 3.0-13.0 | (9) |
| PRO-C3 | N-terminal propeptide of type III collagen (formation marker) | Monoclonal | 2.6-116.0 | 1.8-9.3 | 8.0-12.0 | (10) |
| PRO-C6 | C-terminal of type VI collagen (formation marker) | Monoclonal | 0-8-134.0 | 1.1-5.3 | 3.4-12.4 | (34) |

**Table S1. Target and assay performance characteristics for the ELISA assays specific to each neoepitope.** The upper and lower limits of quantification, as well as intra- and inter-assay variation is also defined for each neoepitope specific assay. LLOQ= lower limit of quantification, ULOQ= Upper limit of quantification.

| **Control Subjects** | | | |
| --- | --- | --- | --- |
|  | **P1NP** | **PRO-C3** | **PRO-C6** |
| **P1NP** | 1.00000 45 |  |  |
| **PRO_C3** | 0.09268 44 | 1.00000 47 |  |
| **PRO_C6** | 0.16857 45 | 0.39667 46 | 1.00000 49 |
| **IPF Subjects** | | | |
|  | **P1NP** | **PRO-C3** | **PRO-C6** |
| **P1NP** | 1.00000 142 |  |  |
| **PRO_C3** | 0.24200 141 | 1.00000 144 |  |
| **PRO_C6** | 0.19174 141 | 0.51438 143 | 1.00000 144 |

**Table S2. Spearman correlations for baseline serum concentrations of synthesis neoepitopes in control and IPF subjects**. Each box shows the correlation coefficient and number of subjects with paired evaluable samples available.

|  | **Univariate HR** | **P value** | **Adjusted HR** | **P value** |
| --- | --- | --- | --- | --- |
| BGM | 1.408 (0.803, 2.469) | 0.2322 | 1.081 (0.570, 2.052) | 0.8111 |
| C1M_01 | 1.838 (1.034, 3.266) | 0.0381 | 1.602 (0.853, 3.008) | 0.1427 |
| C3M_01 | 2.443 (1.386, 4.305) | 0.0020 | 2.346 (1.244, 4.426) | 0.0084 |
| C6M | 2.186 (1.249, 3.824) | 0.0062 | 2.020 (1.085, 3.760) | 0.0265 |
| CRPM | 2.128 (1.207, 3.754) | 0.0091 | 2.339 (1.180, 4.635) | 0.0149 |
| P1NP | 0.756 (0.440, 1.296) | 0.3088 | 0.959 (0.531, 1.733) | 0.8905 |
| P1NP_C1M | 0.731 (0.414, 1.290) | 0.2793 | 0.839 (0.446, 1.580) | 0.5871 |
| PRO-C3 | 1.622 (0.945, 2.786) | 0.0795 | 1.742 (0.940, 3.228) | 0.0777 |
| PRO-C6 | 1.140 (0.672, 1.934) | 0.6280 | 1.415 (0.794, 2.520) | 0.2388 |
| PROC3_C3M | 0.834 (0.488, 1.427) | 0.5085 | 1.021 (0.556, 1.876) | 0.9470 |
| PROC6_C6M | 0.550 (0.318, 0.951) | 0.0324 | 0.611 (0.334, 1.116) | 0.1087 |

**Table S3. Unadjusted and adjusted (for baseline FVC and DLco) univariate analysis of overall survival in relation to 3-month change in neoepitope concentrations**. Data are expressed as mean hazard ratio (HR) with 95% CIs for all thresholds and represent the associated change in mortality risk based on a 2-fold increase in the explanatory value.

| **Spearman Correlation Coefficients Number of Observations** | | | | | | | | |
| --- | --- | --- | --- | --- | --- | --- | --- | --- |
|  | **BGM** | **C1M_01** | **C3M_01** | **C6M** | **CRPM** | **P1NP** | **P1NP_C1M** | **PRO_C3** |
| **BGM** | 1.00000 130 | 0.56667 119 | 0.36018 129 | 0.45151 127 | 0.50657 129 | 0.05869 129 | -0.48759 119 | 0.10003 130 |
| **C1M_01** | 0.56667 119 | 1.00000 121 | 0.33709 121 | 0.56980 119 | 0.39893 120 | 0.00096 121 | -0.72496 121 | 0.04324 121 |
| **C3M_01** | 0.36018 129 | 0.33709 121 | 1.00000 131 | 0.44337 129 | 0.39043 130 | 0.08849 130 | -0.27157 121 | 0.21720 131 |
| **C6M** | 0.45151 127 | 0.56980 119 | 0.44337 129 | 1.00000 129 | 0.48054 128 | 0.07186 128 | -0.37329 119 | 0.06312 129 |
| **CRPM** | 0.50657 129 | 0.39893 120 | 0.39043 130 | 0.48054 128 | 1.00000 131 | 0.10430 130 | -0.23933 120 | 0.27018 131 |
| **P1NP** | 0.05869 129 | 0.00096 121 | 0.08849 130 | 0.07186 128 | 0.10430 130 | 1.00000 131 | 0.45530 121 | 0.28505 131 |
| **P1NP_C1M** | -0.48759 119 | -0.72496 121 | -0.27157 121 | -0.37329 119 | -0.23933 120 | 0.45530 121 | 1.00000 121 | 0.10783 121 |
| **PRO_C3** | 0.10003 130 | 0.04324 121 | 0.21720 131 | 0.06312 129 | 0.27018 131 | 0.28505 131 | 0.10783 121 | 1.00000 132 |
| **PRO_C6** | 0.05845 130 | -0.09033 121 | 0.11509 131 | -0.11485 129 | 0.12537 131 | 0.22182 131 | 0.14010 121 | 0.55443 132 |
| **PROC3_C3M** | -0.14357 129 | -0.12427 121 | -0.36218 131 | -0.21142 129 | -0.00075 130 | 0.17241 130 | 0.19363 121 | 0.70560 131 |
| **PROC6_C6M** | -0.32716 127 | -0.46267 119 | -0.35206 129 | -0.83062 129 | -0.26324 128 | 0.04950 128 | 0.35716 119 | 0.22069 129 |

**Table S4. Spearman correlations for 3-month change in serum concentrations of synthesis and degradation neoepitopes in IPF subjects**. Each box shows correlation coefficient and number of subjects with paired evaluable samples available


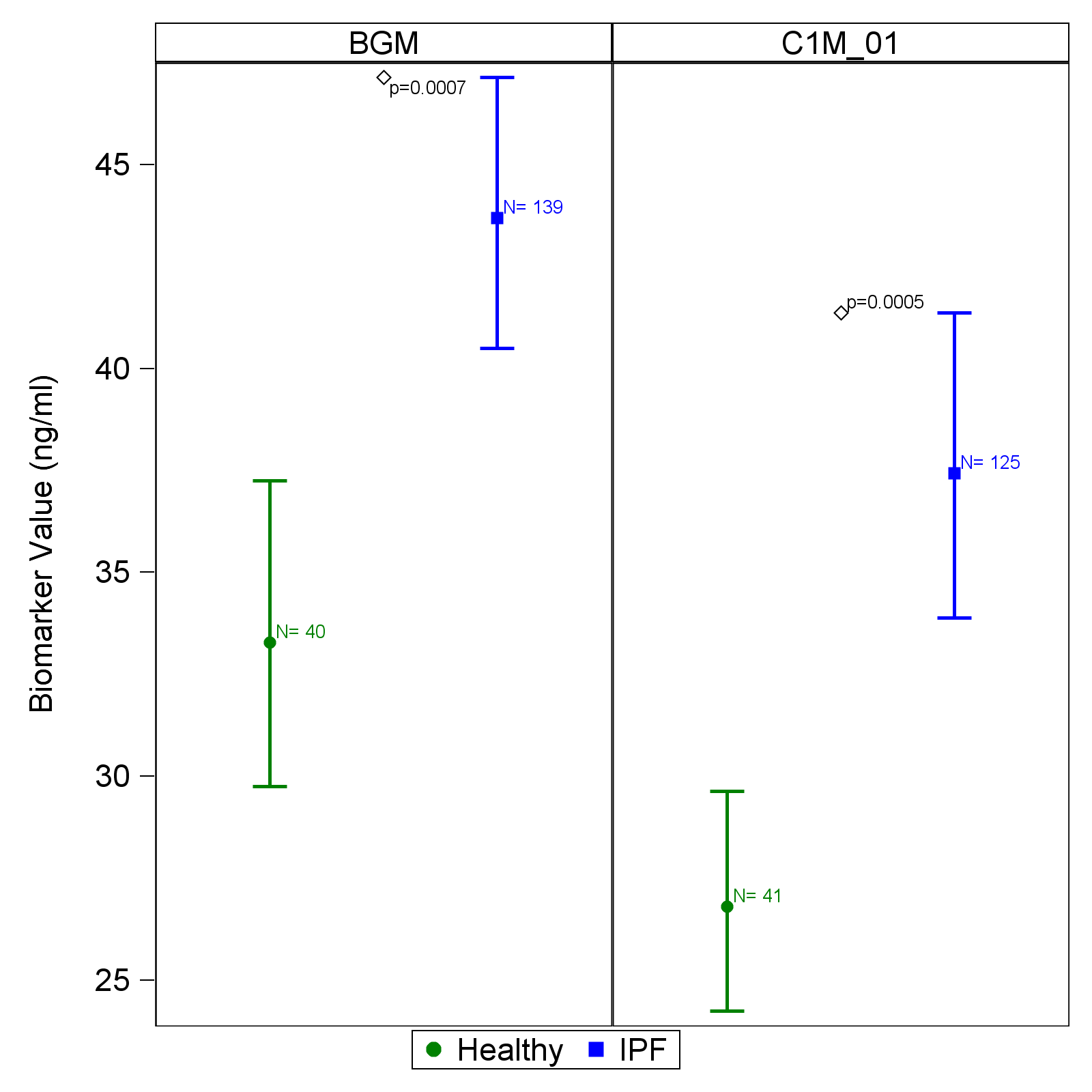

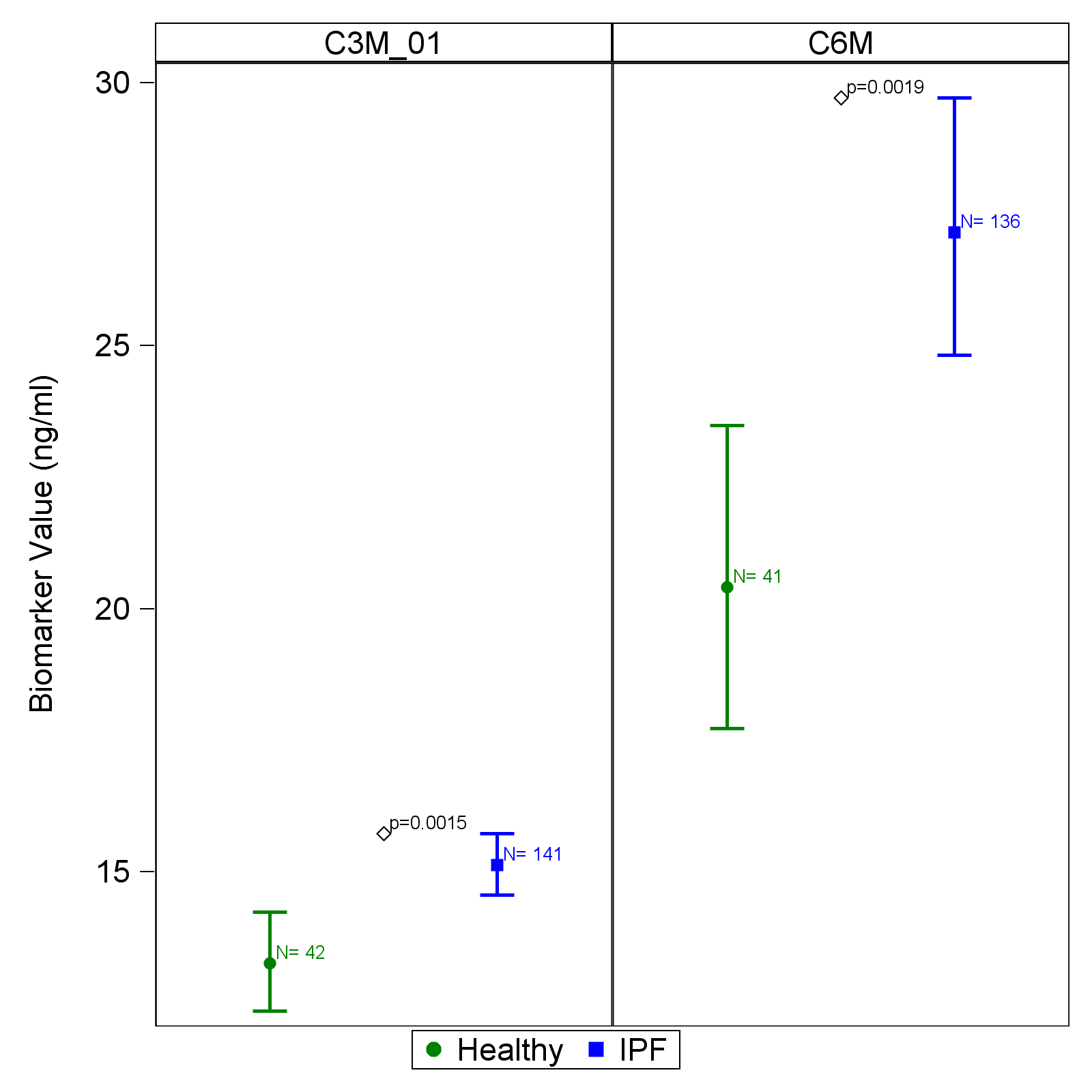

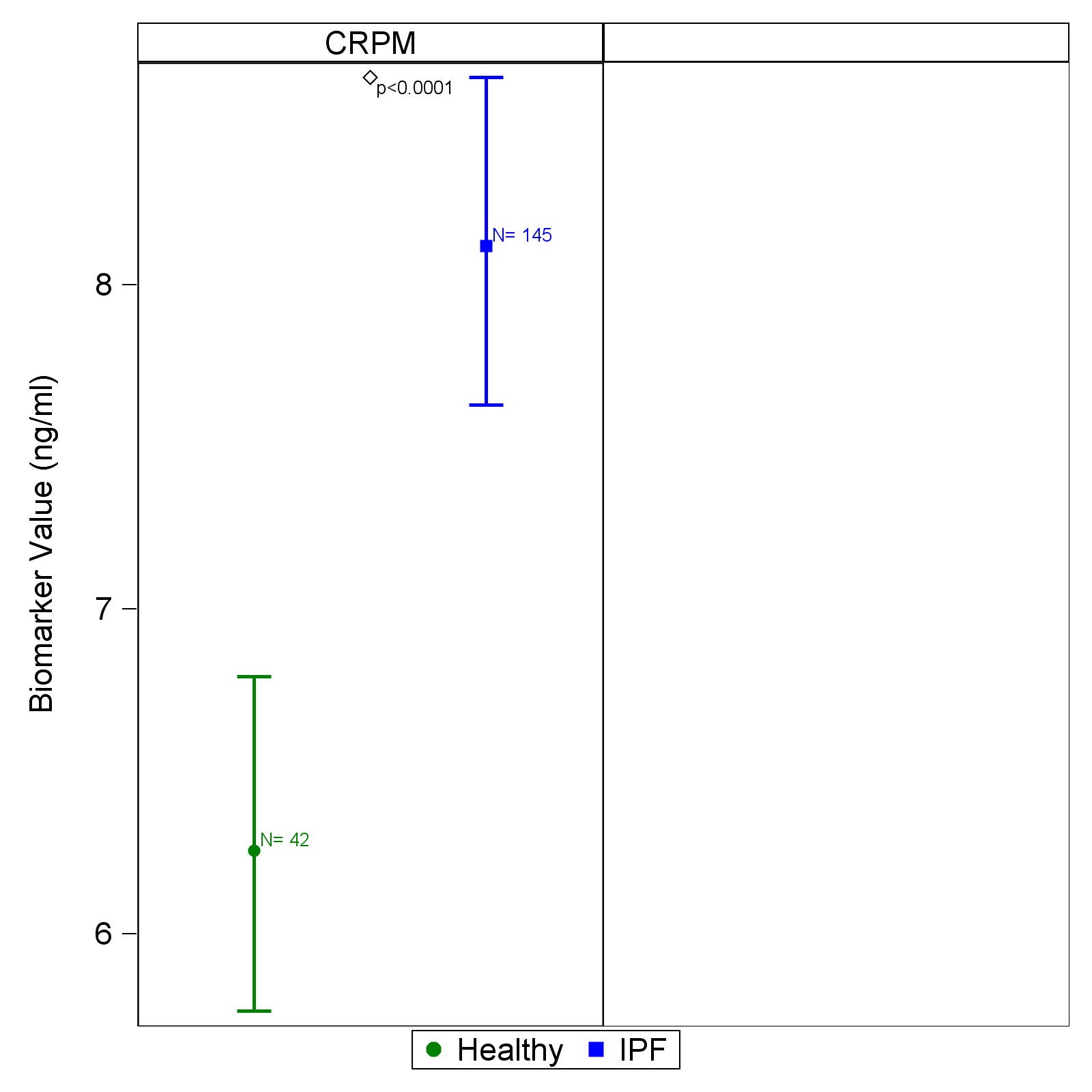


**Figure S1. Baseline comparison of collagen degradation neoepitope (BGM, C1M, C3M, C6M and CRPM) concentrations in healthy controls (n=50) and participants with idiopathic pulmonary fibrosis (n=145).** Plots represent mean and 95% CI (error bars) adjusted for gender.


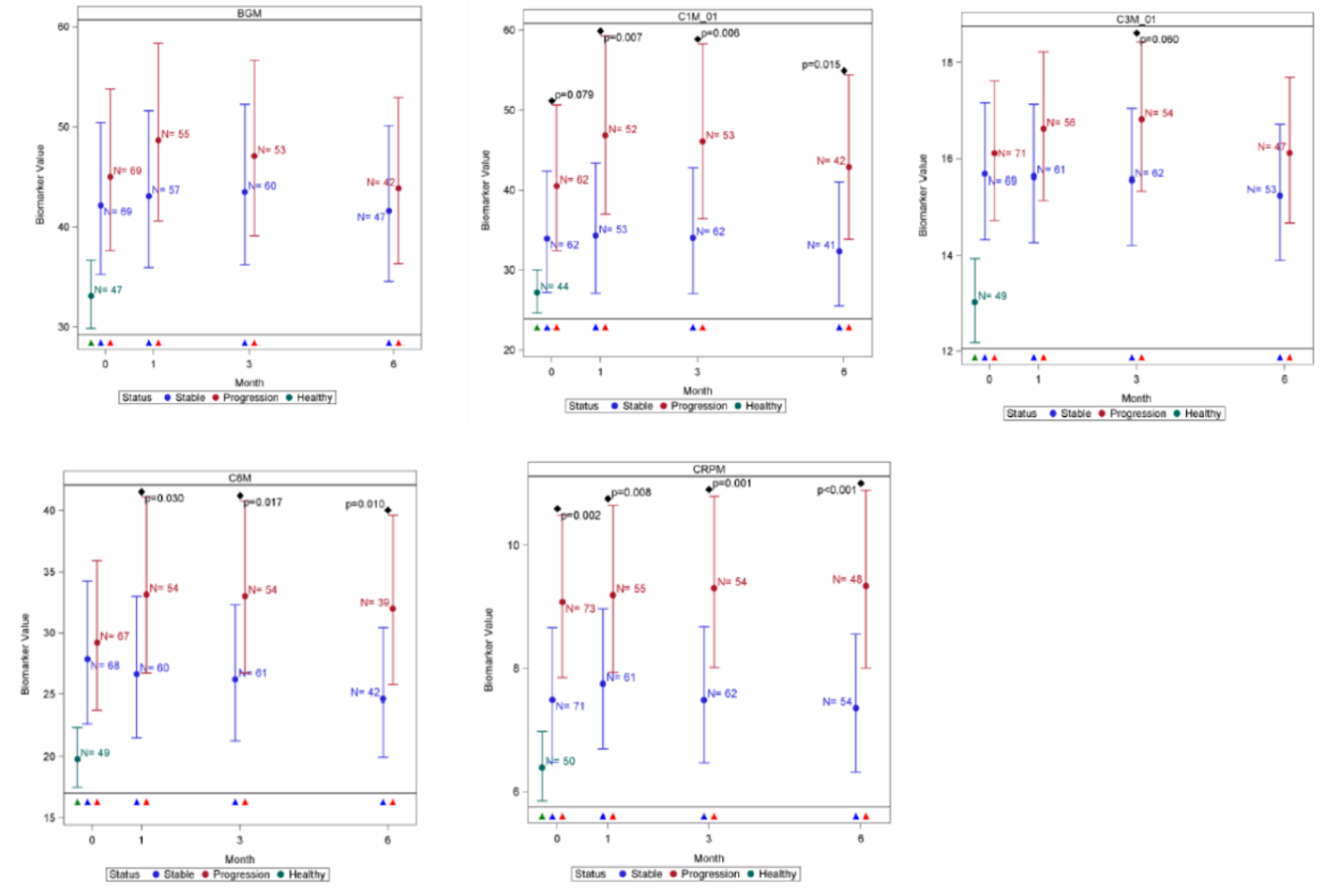


**Figure S2. Comparison of neoepitope concentrations in healthy controls (green) and participants with stable (blue) and progressive (red) idiopathic pulmonary fibrosis at baseline and subsequently at 1-, 3- and 6-months.**Plots represent mean and 95% CI (error bars) adjusted for age, sex, site and smoking status. Disease progression was defined as all-cause mortality or ≧10% decline in forced vital capacity at 12 months. The number of evaluable samples available for analysis at each time point are provided in the graph. P values are provided where significant (p<0.05) differences were observed between stable and progressive disease at a particular time point.

F


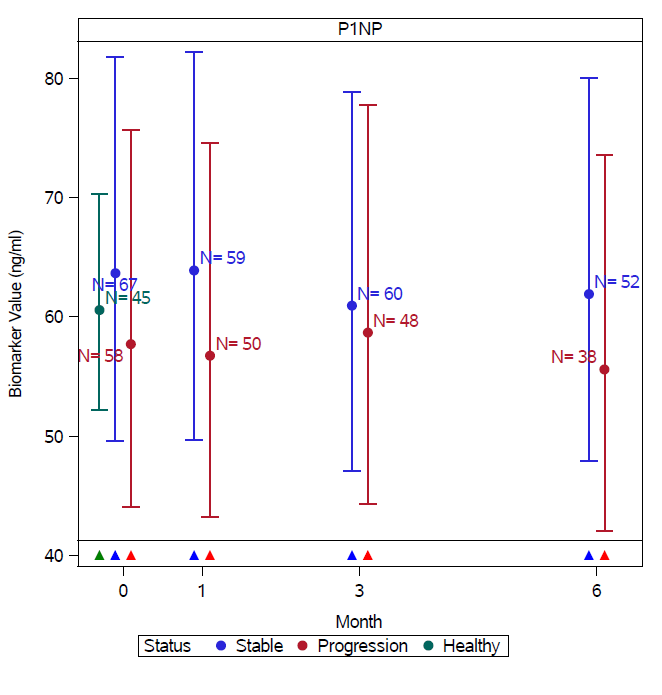

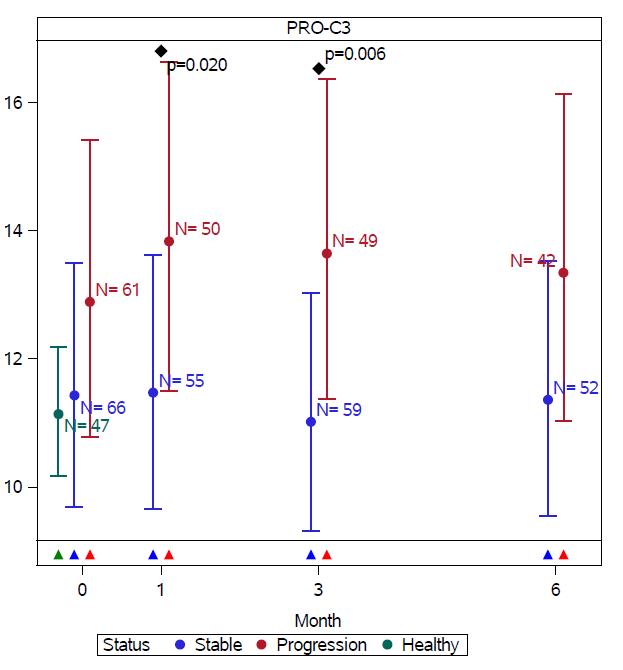

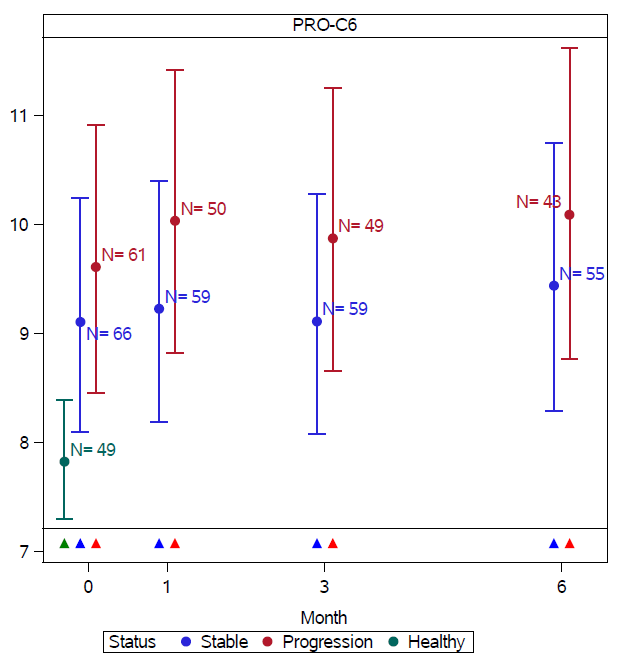


**Figure S3. Comparison of synthesis neoepitope concentrations adjusted for baseline FVC and DLco %predicted in healthy controls (green) and participants with stable (blue) and progressive (red) idiopathic pulmonary fibrosis at baseline and subsequently at 1-, 3- and 6-months.**Plots represent mean and 95% CI (error bars) adjusted for age, sex, site and smoking status. Disease progression was defined as all-cause mortality or ≧10% decline in forced vital capacity at 12 months. The number of evaluable samples available for analysis at each time point are provided in the graph. P values are provided where significant (p<0.05) differences were observed between stable and progressive disease at a particular time point.


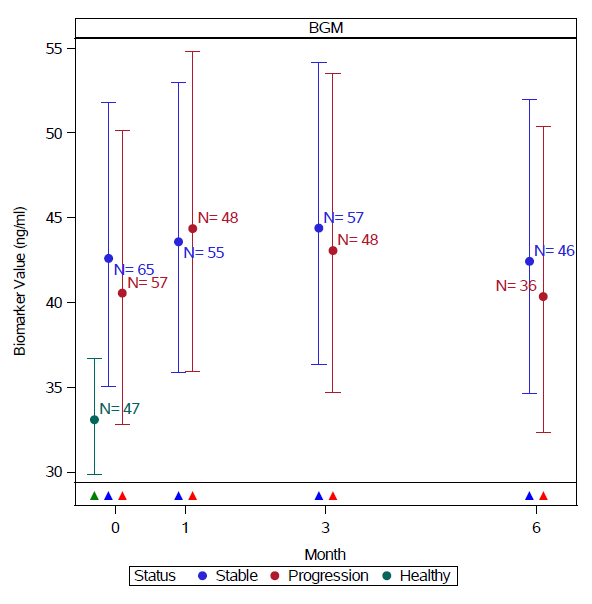

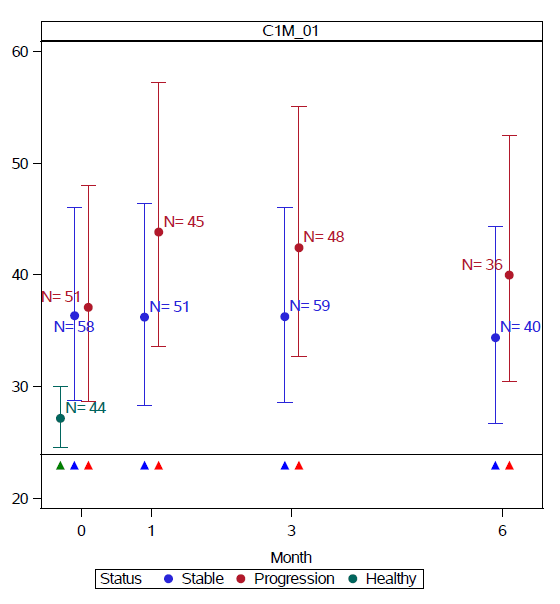

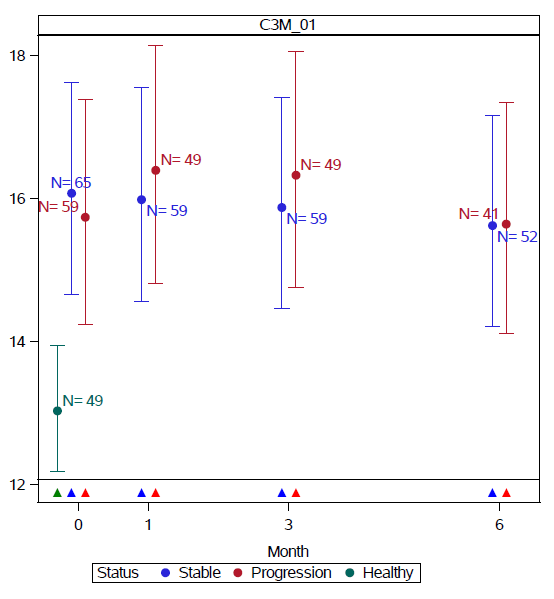

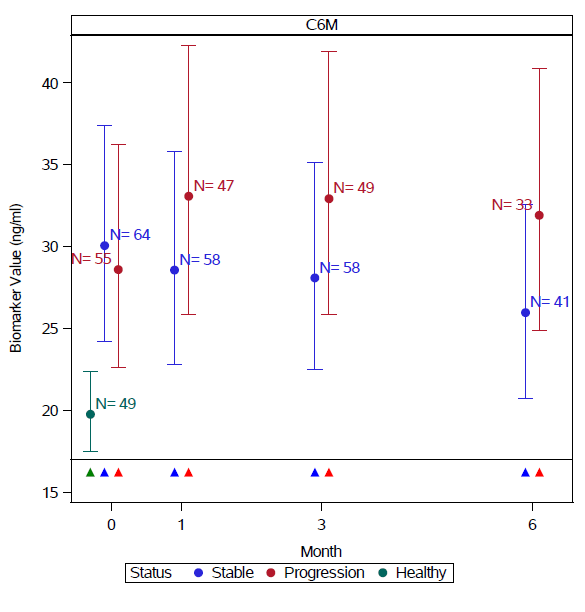

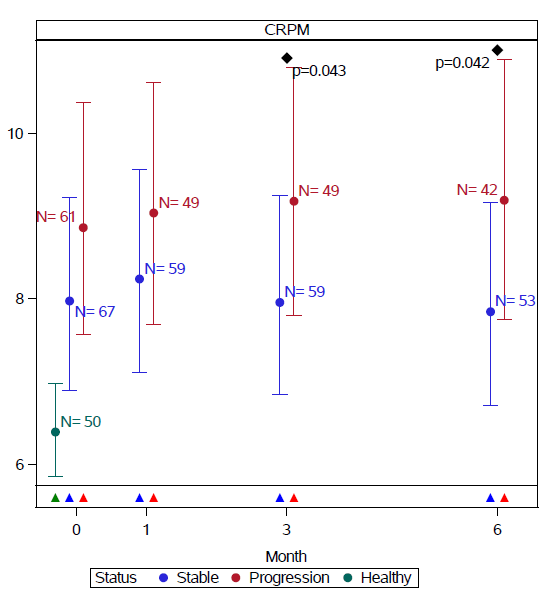


**Figure S4: Comparison of degradation neoepitope concentrations adjusted for baseline FVC and DLco %predicted in healthy controls (green) and participants with stable (blue) and progressive (red) idiopathic pulmonary fibrosis at baseline and subsequently at 1-, 3- and 6-months.**Plots represent mean and 95% CI (error bars) adjusted for age, sex, site and smoking status. Disease progression was defined as all-cause mortality or ≧10% decline in forced vital capacity at 12 months. The number of evaluable samples available for analysis at each time point are provided in the graph. P values are provided where significant (p<0.05) differences were observed between stable and progressive disease at a particular time point.


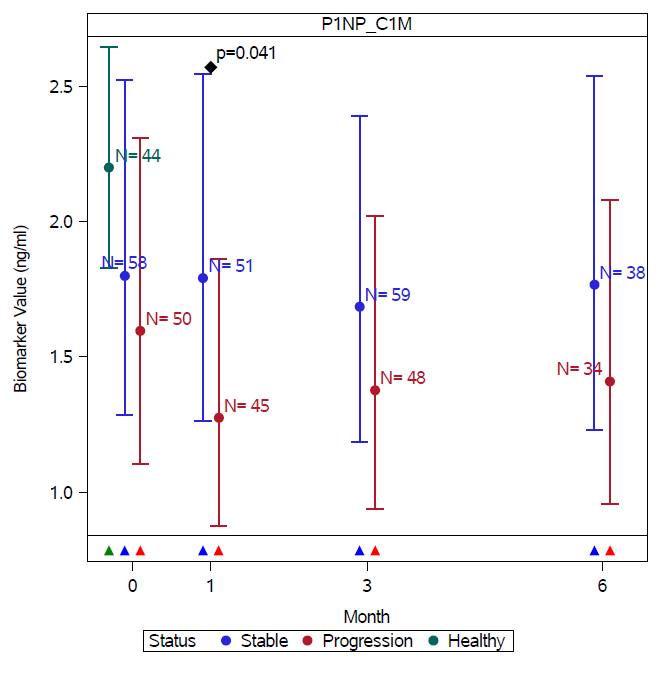

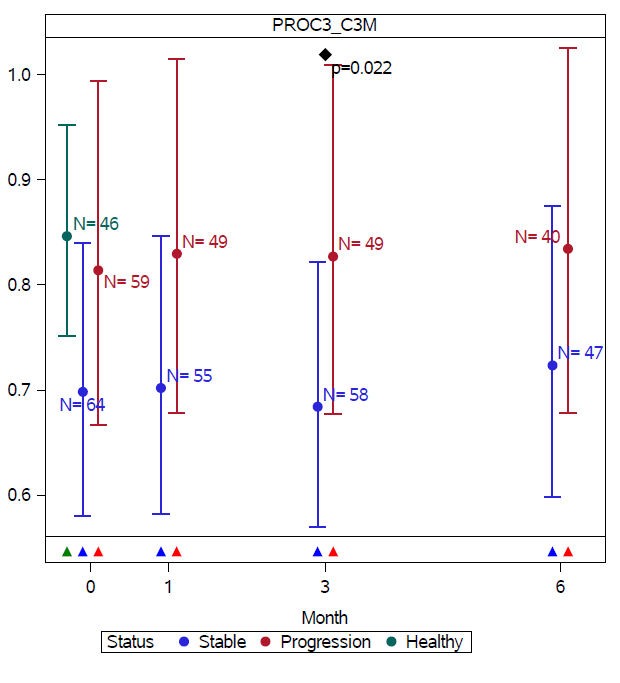

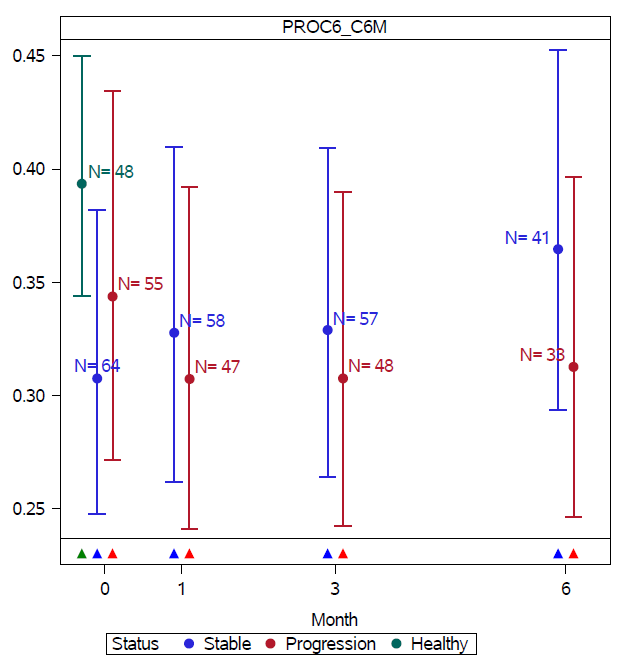


**Figure S5. Comparison of neoepitope concentrations adjusted for baseline FVC and DLco %predicted in healthy controls (green) and participants with stable (blue) and progressive (red) idiopathic pulmonary fibrosis at baseline and subsequently at 1-, 3- and 6-months.**Plots represent mean and 95% CI (error bars) adjusted for age, sex, site and smoking status. Disease progression was defined as all-cause mortality or ≧10% decline in forced vital capacity at 12 months. The number of evaluable samples available for analysis at each time point are provided in the graph. P values are provided where significant (p<0.05) differences were observed between stable and progressive disease at a particular time point
